# Convergence or Divergence? Life Expectancy Patterns in Post-communist Countries, 1959–2010

**DOI:** 10.1007/s11205-017-1764-4

**Published:** 2017-10-28

**Authors:** Christopher J. Gerry, Yulia Raskina, Daria Tsyplakova

**Affiliations:** 1 International Centre for Health Economics, Management, and Policy (CHEMP), National Research University Higher School of Economics, Saint Petersburg, Russian Federation; 2 St. Antony’s College, University of Oxford, 62 Woodstock Road, Oxford, OX2 6JF UK; 3 European University at St Petersburg, Saint Petersburg, Russian Federation; 4 International Laboratory for Economics of Healthcare and Its Reforms, Gaidar Institute for Economic Policy, Moscow, Russian Federation

**Keywords:** Mortality disparities, East–West gap in life expectancy, Post-communist economies, Convergence club methodology, Health crisis, Gender

## Abstract

In the 1960s and 1970s, the countries of Central and Eastern Europe and the Soviet Union experienced an unanticipated stagnation in the process of mortality reduction that was accelerating in the west. This was followed by even starker fluctuations and overall declines in life expectancy during the 1980s and 1990s. We identify statistically the extent to which, since the 1990s, the countries of the post-communist region have converged as a group towards other regional or cross-regional geopolitical blocks, or whether there are now multiple steady-states (‘convergence clubs’) emerging among these countries. We apply a complex convergence club methodology, including a recursive analysis, to data on 30 OECD countries (including 11 post-communist countries) drawn from the Human Mortality Database and spanning the period 1959–2010. We find that, rather than converging uniformly on western life expectancy levels, the post-communist countries have diverged into multiple clubs, with the lowest seemingly stuck in low-level equilibria, while the best performers (e.g. Czech Republic) show signs of catching-up with the leading OECD countries. As the post-communist period has progressed, the group of transition countries themselves has become more heterogeneous and it is noticeable that distinctive gender and age patterns have emerged. We are the first to employ an empirical convergence club methodology to help understand the complex long-run patterns of life expectancy within the post-communist region, one of very few papers to situate such an analysis in the context of the OECD countries, and one of relatively few to interpret the dynamics over the long-term.

## Introduction

Towards the end of the twentieth century the previous trend of improving global health gave way to newly emerged patterns of regional health divergence. Some regions (e.g. South-East Asia) underwent accelerated catch-up, others (e.g. Sub-Saharan Africa) saw their progress stall and in some cases fall backwards, and one region in particular (that containing the countries of Central and Eastern Europe, Russia and Central Asia) experienced unanticipated medium-term stagnation (from the 1960s), followed by major fluctuations and overall declines in life expectancy during the 1980s and 1990s. It is the remarkable dynamics and within-region trends of these former command economies, which form the main focus of this paper.

There are rich and illuminating seams of demographic (Zakharov and Ivanova 1996; Shkolnikov and Cornia 2000; Vishnevsky 2003; World Bank, 2005), epidemiological (Leon et al. 1997; McKee et al. 1998; Bobak and Marmot 1996; McKee and Shkolnikov 2001; Leon et al. 2007; Zaridze et al. 2009), and social science (Shapiro 1995; Shkolnikov et al. 1998; Cockerham 2000; Cornia and Paniccià 2000; Davis 2000; Brainerd and Cutler 2005; Gerry 2012) literature examining the nature of these trends and fluctuations. In Russia, perhaps the most extreme and certainly the most well-documented case, life expectancy for males collapsed from 64.2 in 1989 to 58.3 in 1995. These dramatic events were not though restricted either to Russia or to the post-communist period. Right across the so-called ‘mortality belt’—the western countries of the former Soviet Union^1^– life expectancy for both males and females had declined from the 1960s and then fluctuated considerably *both* during the 1980s (the late Soviet period) and the 1990s (the ‘transition’ period). However, while similar economic and political disruption occurred across the region in the early 1990s, the post-communist countries bordering the mortality belt from either side, suffered much less substantive decreases in their life expectancy during this period. Moreover, since the 1990s, a further divergence has developed, with parts of the mortality belt (e.g. the Baltic countries) now converging on the dynamics of Western OECD countries in terms of life expectancy, while the remainder remain in stagnation.

The literature referred to above has predominantly focused on the important task of trying to understand and explain the unprecedented fluctuations of the 1990s, often in the context of the longer-term decline. There is agreement that there were indeed uniquely sharp fluctuations in life expectancy during the 1980s and 1990s and that these effects were felt more acutely in the so-called mortality belt. Additionally, though much remains disputed, there is also consensus that the increased dangerous use of alcohol (including surrogate alcohol) and the stress associated with socioeconomic upheavals across the region was closely associated with these fluctuations.

A distinctive strand of research covering the post-communist region has followed the path of Brainerd and Cutler in drawing attention to emerging intra-region patterns of divergence and convergence. Meslé (2004), first identifies the potential split of the mortality belt and thereafter the literature (e.g. Stuckler et al. 2009) typically takes as its point of departure the stylized fact that there are two sub-regions emerging from the transitional process itself: the Central and East European countries (CEE) and Baltics, consisting of those countries that have subsequently joined the European Union, and the remaining former Soviet Union (FSU) countries, that lag behind. While this literature generally doesn’t explore the nature or determinants of these groupings, it has sparked an important debate concerning the extent to which the reform policies of the early 1990s were the cause of heterogeneity in health outcomes within the post-Communist region (Earle and Gehlbach 2010; Gerry 2012; Stuckler et al. 2012).

The stark life expectancy experiences of the post-Communist world have drawn attention to a more generalised global phenomenon of increasing intra-country and between country inequality in life expectancy. Correspondingly, a new literature, examining mortality trends across large swathes of the globe has emerged (Caselli et al. 2002; Wilson 2001; Moser et al. 2005; Smits and Monden 2009; Edwards 2011) establishing the following: there is great variation in life expectancy over time and place (Smits and Monden 2009); the convergence that had characterised global life expectancies through to the 1980s, reversed thereafter, largely due to increasing divergence in adult mortality (Moser et al. 2005); total inequality is increasingly due to between-country variation, particularly among high income countries (Edwards 2011) and; relatively little is known about the sources of between or within country inequality in life expectancy, or its relation with other socioeconomic inequalities (Wilson 2001; Edwards 2011).

These empirical descriptions of the changing pattern of global life expectancies have prompted a more analytical stream of empirical research (Timonin et al. 2016). It is to this latter strand of literature that we seek to contribute. We explore the nature of sub-group patterns in health outcomes, over a period (1959–2010) which captures the history of 30 countries, including 11 transition countries, and which views them within a broader—OECD wide—health dynamic. Crucially, unlike in the approach of Timonin et al. (2016), our methodology allows for life expectancy convergence patterns to be defined by the data and so the groupings which emerge are not pre-defined according to a priori beliefs but are a product of statistical testing.

In this context, our main contributions are fourfold: (1) we are the first to identify statistically whether there are multiple steady-states (what the literature knows as ‘convergence clubs’) emerging among the transition countries either separately or alongside western countries; (2) on the basis of theses statistical findings, we document the extent to which transition countries have formally converged towards the more mature market economies; (3) following Timonin et al. (2016), but using our methodology, we explore these questions for a range of age and gender specific life expectancies; and (4) we add to the important body of literature that stems from the pioneering work of Vallin and Meslé (2004), (re)theorising global health and demographic transitions.

We make several important claims. First, there has been no major ‘east–west’ convergence in life expectancy in the post-Communist period. On the contrary, there has been substantial divergence within the set of transition countries, often into 3 or 4 groupings, headed by the Czech Republic, which has converged towards western levels of life expectancy, and with Russia, Ukraine and Belarus, typically found in the lowest group. The remaining Baltic and CEE countries form groups that lie between these two extremes, but where the composition is gender and age specific. Indeed, gender and age transpire to be important: the heterogeneity that we observe among these countries is not a product of mortality differentials at young ages as there is a single grouping, containing all OECD and transition countries, for life expectancy below age 16; and for females in particular, the number of clubs increases over time, and at higher ages, and contains more mixed (OECD and transition) country clubs. This latter finding provides support for the Vallin and Meslé (2004) theory of convergence and divergence patterns in advanced economies.

The paper is structured as follows. In Sect. 2 we flesh out in more detail the emerging literature on convergence and divergence in life expectancies. In Sect. 3, we outline our two-part econometric strategy, based on that of Phillips and Sul (2007), and give details of the data we use in applying this methodology. Section 4 presents the two sets of convergence test results. Section 5 provides a concluding discussion, identifying strengths and weaknesses, as well as future research directions.

## Literature Review

We referred above to the rich set of literature exploring the 1990s ‘mortality crisis’ from different perspectives as well as to the more recent, and correspondingly smaller, strand of research examining intra-region convergence and divergence. To date there has been little attempt to catalogue analytically the extent to which different groups of these countries, and different sub-groups of the population within these countries, are converging (or not) on different equilibrium trends in a global or multi-region context. This is the gap in the nascent literature, reviewed below, that we are addressing.

Mayer-Foulkes (2001) was one of the first authors to explore the importance of club convergence (i.e. multiple steady-states) in life expectancy. He argued persuasively that the club convergence approach can serve as a theoretical framework to capture the idea of multiple states of economic development and that understanding this is crucial for policy making because it helps us to understand the patterns of transition between different stages of economic development. Drawing on the five-yearly (1962–1997) balanced panel data of Easterly and Sewadeh (2001), covering 159 countries, he employed a fixed-effects convergence model with dummies for three hypothetical clubs. He found that an approach allowing for multiple steady states offers a better fit for these data: countries with life expectancy less than the sample median in both 1962 and 1997 formed the first club; countries with life expectancy less than the median in 1962 and higher than the median in 1997 formed the second club; and the remaining countries formed the third ‘high life expectancy’ club.

McMichael et al. (2004) also identified three clubs of countries for the period 1950–2005: countries with broadly positive trends in life expectancy; countries experiencing relative stagnation; and countries experiencing a negative trend. More recently, Canning (2012) discussed the existence of two convergence clubs in life expectancy and applied a regime switching model, in which the probability of a particular regime depends on initial life expectancy. He used a sample of 174 countries during the period 1970 to 2005 and found no support for overall convergence in life expectancy, instead finding that two distinctive clubs emerged.

Chung and Muntaner (2007) adopted a different approach, studying the parameters of health outcomes (infant mortality and low birth weight) in 19 wealthy countries. Their hypothesis, tested over the period 1960–1994, was that population health indicators in wealthy industrialized countries are “clustered” around welfare state regime types. They defined four different types of welfare state regime (Social Democratic, Christian Democratic, Liberal and Wage Earner) and estimated conditional hierarchical models in which the welfare state type acts as a fixed effects indicator at the country-level. They find that countries do indeed exhibit distinctive levels of population health by welfare state types.

Edwards and Tuljapurkar (2005), among the first to emphasize the importance of taking into consideration the distribution of age at death across countries, found convergence in infant mortality and average age at death among advanced countries since 1960. However, as in Moser et al. (2005), they also find considerable evidence of heterogeneity in the patterns of adult life expectancy.

Vallin and Meslé (2004) and Meslé and Vallin (2011), in their invaluable review of historical trends in mortality, identified three historical ‘waves’ from the eighteenth century to the present. The first wave, ‘Pandemic Receding’ spanning from the end of the eighteenth century through to the middle of the twentieth century, mirrors Omran’s (1971) theory of “epidemiologic transition”. Initially, developed countries experience rapid increases in life expectancy as a result of the successful fight against infectious and respiratory diseases, which takes them ahead of less-developed countries, thus producing a global divergence in life expectancy, before the catching up of less developed countries generates convergence. Then follows the ‘Cardiovascular Revolution’, in which deaths due to CVD recede in the latter half of the twentieth century, as first identified by Olshansky and Ault (1986). Meslé and Vallin then focused on newer processes of divergence-convergence based on (1) the capacity of society to achieve health gains through improved social conditions, behavioural changes, and health policies; and (2) the tendency for developed countries to make significant life expectancy gains at old age.

Finally, a more recent piece (Timonin et al. 2016), offers a complimentary analytical approach for understanding heterogeneity in life expectancy and applies it in the context of the countries of Central and Eastern Europe and the former Soviet Union. Timonin et al. use population-weighted cross country variance as their measure of intercountry disparity. They define three ‘developed’ geopolitical groupings—established market economies (EME), Central and Eastern Europe (CEE) and the former Soviet Union (FSU). They describe the nature and extent of the growth in life expectancy variance and present a tool for decomposing this variance into between region and within region variance, while also accounting for gender and age specific patterns. They find that, as of 2010, heterogeneity in life expectancy outcomes across these developed countries was greater than in 1970. They attribute a substantive core of this rise to between-group variance reflecting the excess mortality among the working age (principally males) that has defined the East–West mortality divide in Europe during the last three decades (Meslé 2004). In contrast, they find that, over time, the within-group variance was increasingly dominated by heterogeneity in female life expectancy, which they argue stems from differential country-by-country success in reducing female mortality at older ages (Vallin and Meslé 2004).

While, in combination, this literature begins to identify important trends in patterns of global life expectancy, there are two important empirical fault lines on which we build in this paper. First, the approach to identifying the number of clubs is largely descriptive and based on either visual analysis of trends and parameters or on a priori speculation of appropriate geopolitical or economic groupings. Second, except for Edwards and Tuljapurkar (2005) and Meslé and Vallin (2011), the studies to date tend to focus on life expectancy at birth and/or on total mortality. However, as Meslé and Vallin (2011) argue, mortality trends are determined by larger forces at work within society and that impact upon different ages according to the stage of overall development. The methodological contribution of our paper is defined through its responses to these two weaknesses. That is, first, we use a robust methodology to identify statistically the convergence clubs in the data. Second, we do so in a manner that allows us to explore this question for different age and gender groups.

## Data and Econometric Methodology

### Data

We use the life expectancy data from the Human Mortality Database (HMD),^2^ covering 30 countries (including 11 transition countries) between 1959 and 2010. These data allow us to access high quality mortality statistics for the longest time span of life expectancy, including among older age groups, which contribute significantly to the patterns we observe in the data. The HMD also allows us to access data, by age and gender, for a panel of countries including a subset of transition economies. The 11 transition countries for which there are data represent three stylised sub-regions: (1) Central and Eastern Europe (CEE), represented by Bulgaria, Czech Republic, Hungary, Poland and Slovak Republic; (2) the Baltic Countries (BC) of Estonia, Latvia and Lithuania; and (3) the Commonwealth of Independent States (CIS) countries of Belarus, Russia and Ukraine.

In our analysis, we use the following life expectancy measures (separately for male and female): (1) life expectancy at birth (denoted by e0); (2) average number of years lived between ages 0 and 15 among those surviving to age 0 (denoted by 15e0); average number of years lived between ages 15 and 65 years among survivors to age 15 (denoted by 50e15); and life expectancy at age 65 (denoted by e65).^3^

### Descriptive Analysis

**Fig. 1:**
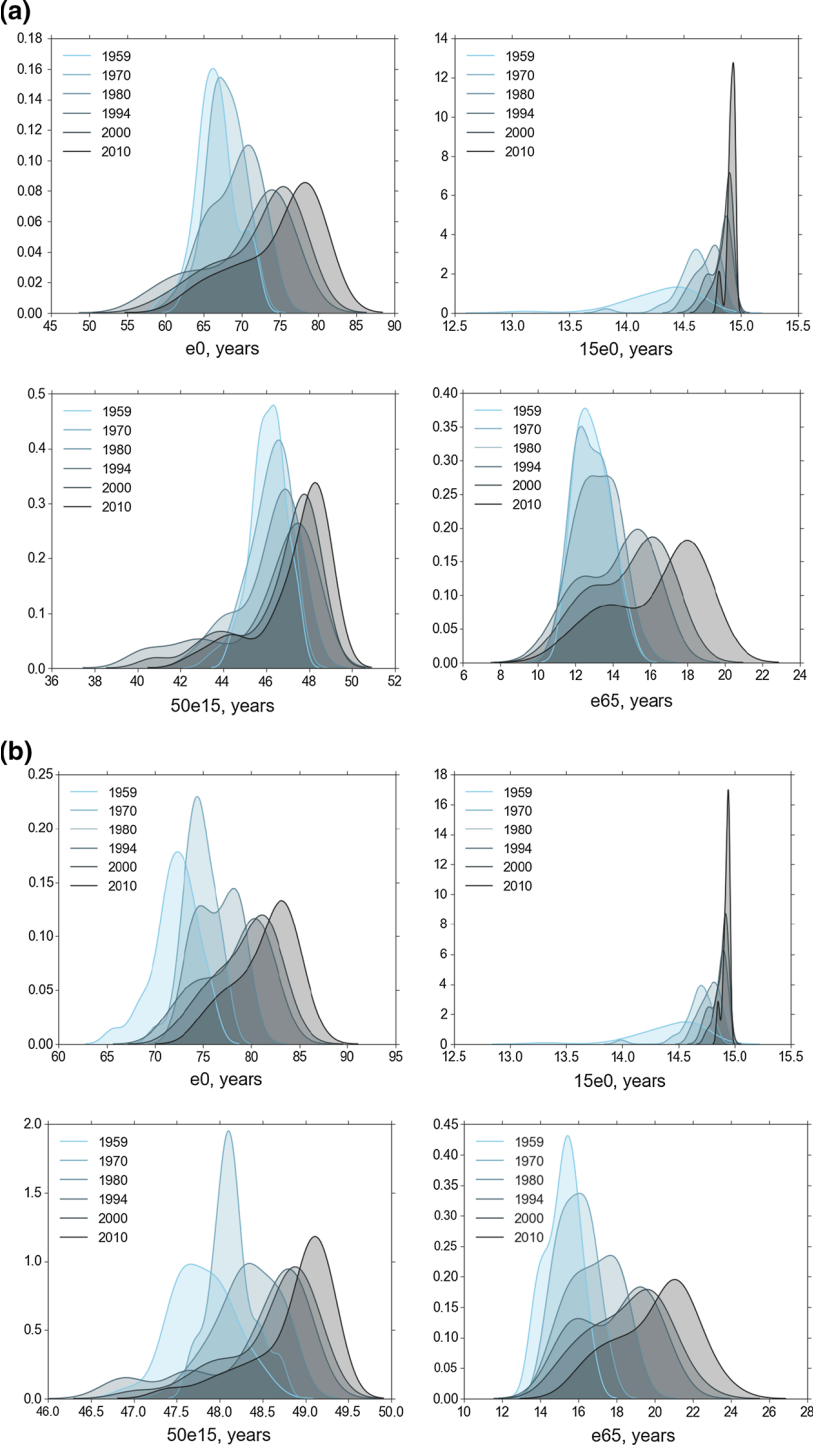
**a** Distribution of male life expectancy. **b** Distribution of female life expectancy. *Note* for Figs. 1 and 2: male/female life expectancy distributions: e0, 15e0, 50e15, e65; respectively plotted (kernel density estimation) for the years 1959, 1970, 1980, 1994, 2000, 2010

As with the studies reviewed earlier, there is much that can be learnt through descriptive and visual analysis and so we start by examining the life expectancy distributions in our data. From the kernel density estimates in Fig. 1a (male) and b (female), a visible divergence, *alongside* overall increases in life expectancy (darker shading) since 1980, is clear in all cases other than for 15e0, the average number of years lived between 0 and 15, for which the increase takes place without the divergence. This suggests, consistent with other literature, that the global inequalities we are observing are driven by heterogeneity within older populations. In suggesting higher life expectancy variance by age and gender these two figures offer the first hint that there may be multiple life expectancy ‘clubs’ emerging. Indeed, there is not only a widening of the distribution but also the formation of a ‘second peak’ at all ages—a bimodal pattern which is especially visible for male life expectancy at age 65.

**Fig. 2:**
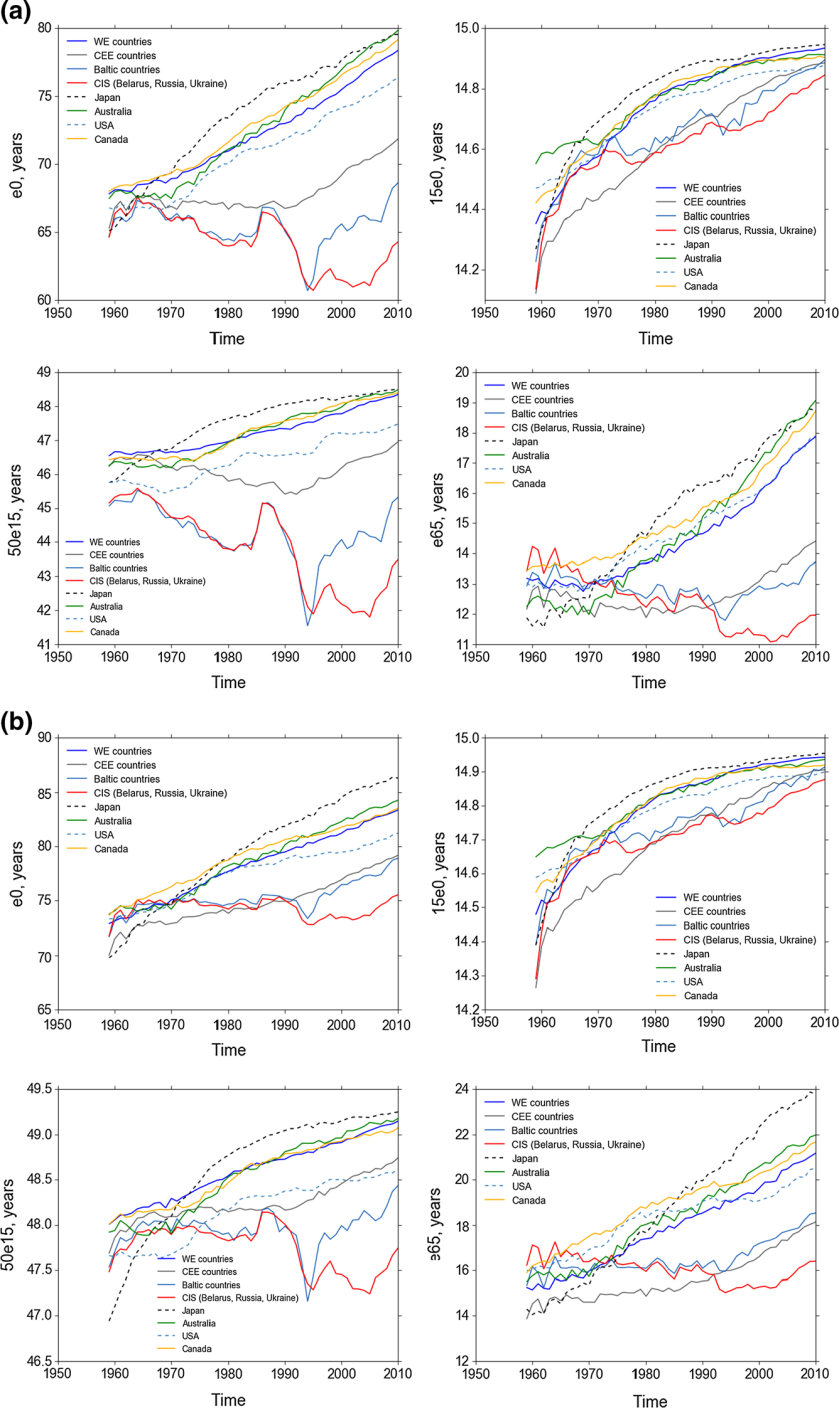
**a** Mean male life expectancy. **b** Mean female life expectancy

Turning now to our interest in the possible regional blocks that have emerged, Fig. 2a (male) and b (female) clearly demonstrate how life expectancy for both males and females in the post-Communist region has diverged from the developed country trend since the start of the 1970s, with Russia, Belarus and the Baltic countries experiencing a dramatic decrease, particularly among males, at all ages (once again with the exception of the 15e0 groups) during the early part of the 1990s. The dynamics of Russia, Ukraine, Belarus and the Baltic countries clearly differ from those of the CEE countries. In the former there were several periods of sharp decline in life expectancy at different ages, while in CEE, the stagnation of the 1960s and 1970s has given way to a gradual rise in life expectancy. All three transition regions have recently seen a significant improvement in life expectancy, and this has particularly benefitted the Baltic States, who have converged closer to the CEE life expectancies, particularly for life expectancy at age 65.

### Empirical Methodology

To formally identify the equilibrium ‘clubs’ in these data, without the need for making a priori assumptions, we employ a regression-based methodology (Phillips and Sul 2007) which examines whether the variance in life expectancy across cross-sectional units decreases over time. By decomposing the variable of interest, *X*_*it*_ (life expectancy in our case), into a common factor *μ*_*t*_ and unit specific factor loadings *δ*_*it*_, Phillips and Sul (2007) offer the following representation of a time varying factor model:

$$X_{it} = \delta_{it} \mu_{t} .$$

where *μ*_*t*_ represents a common trend component over time and *δ*_*it*_ represents the relative share in *μ*_*t*_ of country *i* at time *t*. Thus, *δ*_*it*_ is a measure of the individual distance between the common trend component *μ*_*t*_ and *X*_*it*_. The contribution of Phillips and Sul (2007) is to develop an econometric test of convergence for the time varying idiosyncratic components. Underlying this test is the hypothesis that $\delta_{it} \to \delta \;\;{\text{for}}\;{\text{some}}\;\delta \;{\text{as}}\;t\,\; \to \infty \;{\text{and }}\alpha \ge 0,$ where α represents the speed of convergence. This approach, has the useful property, of not requiring any assumptions concerning trend stationarity or stochastic nonstationarity in either *X*_*it*_ or *μ*_*t*_.

At the core of the empirical methodology is the calculation of a ‘relative transition parameter’ *h*_*it*_ which is constructed to measure a country-specific component that varies over time in relation to the panel average at time *t* and to capture the relative departure of a country’s data series from the common growth path:

$$h_{it} = \frac{{X_{it} }}{{\frac{1}{N}\sum\limits_{i = 1}^{N} {X_{it} } }} = \frac{{\delta_{it} }}{{\frac{1}{N}\sum\limits_{i = 1}^{N} {\delta_{it} } }}$$

If panel units converge, the relative transition parameters converge to 1 and in this case, the cross-sectional variance of *h*_*it*_, denoted by *H*_*t,*_ converges to zero. In this context, Phillips and Sul (2007) have shown it is possible to empirically evaluate the null hypothesis of convergence using a *log t* regression:

$$\log \left( {\frac{{H_{1} }}{{H_{t} }}} \right) - 2\log L(t) = a + b\log t + u$$

where *L(t)* = *log(t* + *1)* and the fitted coefficient of log *t* is $\hat{b} = 2\hat{\alpha }$, where $\hat{\alpha }$ is the estimate of the speed of convergence. Following Andrews (1991) a one-sided *t* test is applied and HAC (Heteroskedasticity and Autocorrelation Consistent) errors are used to test the inequality of the null hypothesis *α* ≥ 0.

However, while this describes the core logic of the methodology, rejection of the null for the aggregate panel does not imply the absence of club convergence. Phillips and Sul (2007, 2009) therefore extend their own methodology and develop an algorithm for testing club convergence, based on a stepwise and recursive application of *log t* regression tests to subsamples of the data. With reference to our empirical example, the procedure consists of the following steps: order the entire panel from the most successful to the least successful country according to life expectancy; select the core group of a club based on the *log t* regression test; sequentially add new countries to the core group and repeat the *log t* regression test to confirm their convergence with the core group; repeat the *log t* regression test in this manner for all countries remaining in the sample; once complete, repeat the process for the remaining countries yet to be allocated a club. If at the end of the process, some countries are without a club, they are said to diverge. “Appendix 1” provides further technical details on the *log*-*t* regression methodology and description of the club convergence procedure.

In our case, as shown in the next section, our analysis establishes a robust sense of the convergence process by 2010. However, it is quite possible that during the period under review there is transitioning over time between clubs; for example, as the upper members of a lower club catch up with the lower members of a higher club. This is especially likely after the 1990s due to the mortality crisis in the transition countries and so to investigate this question, and as a further sensitivity test, we implement the recursive club convergence approach for the four male and female life expectancy categories in our data, for the period 1990–2010. That is, we effectively generate an additional 20 club convergence results for the period since transition began (1990–2010), using the initial conditions we observe in 1959.

## Results

### End-of-sample Convergence Analysis

One of the questions at the heart of our research pertains to whether there is statistical evidence that the former command economies of Eastern Europe have converged on the health profiles of the Western European countries. Table 1 below provides an overall summary of the allocation of clubs by gender and by age cohort. The first important thing to note is that the allocation of countries to clubs is successful. Other than for the youngest group (average number of years lived between ages 0 and 15 among those surviving to age 0), there are at least 3 groups in each category with distinctive sets of western as opposed to transition clubs.

**Table 1:** Summary of convergence club allocation

	Number of clubs	Number of western clubs (diverging countries in brackets)	Number of transition clubs (diverging countries in brackets)
e0, Male	5.0	1.5 (0)	3.5 (2)
e0, Female	4.0	1.0 (1)	3.0 (0)
15e0, Male	1.0	0.5 (0)	0.5 (0)
15e0, Female	1.0	0.5 (0)	0.5 (0)
50e15, Male	4.0	1.5 (1)	2.5 (4)
50e15, Female	6.0	3.0 (0)	3.0 (3)
e65, Male	3.0	0.5 (1)	2.5 (2)
e65, Female	5.0	2.5 (2)	2.5 (0)

The fractional number of clubs reflects the presence of mixed clubs containing at least one Western and one transitional country

Table 2 presents the summary of the allocation, respectively for: life expectancy at birth, life expectancy at age 15, for those surviving to 0; life expectancy at age 65 for those reaching age 15; and life expectancy at age 65. These results are presented separately for males and females. Ignoring the second category for now, it is no surprise to see that on average, no matter the gender or age cohort, western country clubs dominate the top of the distribution and transition country clubs dominate the lower life expectancy clubs, but also that the latter tend to fall into more clubs than the former, suggesting that some countries of the transition region are stuck in low-level equilibria, while others are beginning to catch up with western advanced economies. It is also worth observing that, among the western countries, older female age groups tend to fall into more separate clubs than either young female groups or any male groups. This is consistent with the Vallin and Meslé (2004) hypothesis that female mortality exhibits greater heterogeneity due to the differential progress made by countries in handling the transition from deaths caused predominantly by cardiovascular disease to deaths due to multiple chronic diseases.^4^

**Table 2:**
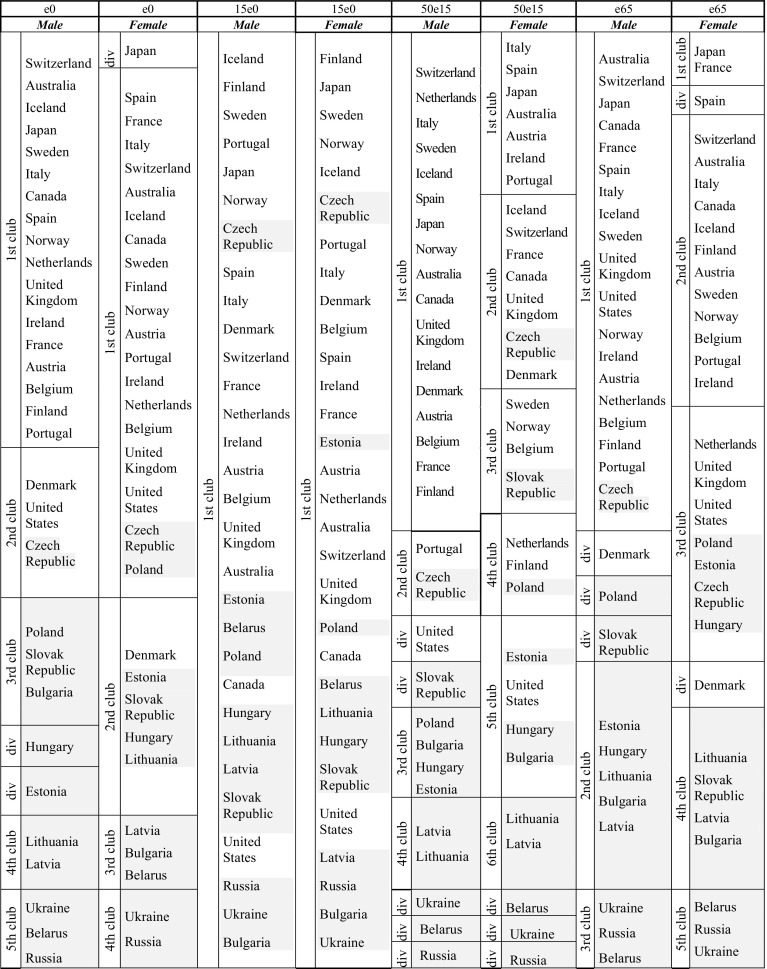
Convergence club membership

Transition countries shaded, within each club countries are listed in descending order of 2010 life expectancy

*div* diverging country

Note that club ranking is important because it indicates the position of convergence clubs *relative to each other*. A change in club position does not necessarily mean a change in the equilibrium pattern of convergence for a given country. For example, if countries with higher life expectancy form two different clubs instead of one, as with female life expectancy at age 65, where France and Japan form their own unique club, then the relative rankings are all shifted downwards by one place. In this context, it is also important to note that not all countries converge on a ‘club’ and may be left ‘alone’ as clubs split over time. Typically, it is the transition countries, with 11 diverging cases, that don’t converge as they are either moving between clubs or, in the case of Russia, Ukraine and Belarus, are in the bottom 3 places of the rankings but are not sufficiently close in trend to one another. There are 4 diverging countries from the OECD group—the United States for the middle-age male group, Denmark for elderly male and female life expectancy, and Spain for elderly female life expectancy. In the latter case this reflects Spain moving out of the second OECD group, but not yet having reached the club comprising of France and Japan. In the former cases, this reflects that the US, with its fragmented and partial health system, and Denmark, with its long history of heavy tobacco consumption, have fallen behind the OECD dominated groups but have not been caught up by the transition dominated groups.

Turning now to the life expectancy categories. For *life expectancy at birth*, the OECD countries are divided into two clubs for men and one club for women. In the latter case, the Czech Republic and Poland have caught up with the leading group (with Denmark falling into the second club), while in the male case, the Czech Republic (male life expectancy, 74.43) has entered the second club, alongside Denmark and the United States, while Poland remains in a third club with Bulgaria and the Slovak Republic. There is far greater heterogeneity in life expectancy at birth among the transition countries, with 3 (2) exclusive male (female) clubs in addition to the presence of countries in 1 (2) mixed club(s). For males, clubs 3, 4 and 5 respectively take us from the more CEE based countries, through the less developed Baltic Republics and on to the CIS group, of Russia, Ukraine and Belarus, with the lowest life expectancies. For female life expectancy in transition countries the pattern is largely repeated.

Reflective of the rapid progress made in reducing infant mortality towards western levels, it comes as no surprise that the second category—*average years lived by those surviving to zero*—produces one club for both males and females. Within those clubs the transition countries are predominantly towards the bottom, with Russia, Bulgaria and Ukraine making up the bottom 3 in both clubs. However, the Czech Republic is towards the top of both clubs, while the United States, Canada, Australia and the United Kingdom fall in the lower half of each club.

Some of the most interesting traction is to be found in the third category, representing the *average life expectancy of those that reach age 15*. For OECD males, there are two clubs: one very large exclusive one, comprising of 17 OECD countries and one smaller one reflecting Portugal trailing behind the core club and having been caught up by the Czech Republic. Among women there is much more heterogeneity, with the 18 OECD countries spread across 4 clubs, a small exclusive one of 7 members, followed by a second club with 6 members and the Czech Republic, followed by two further separate clubs, joined respectively by Slovak Republic and Poland. The transition countries are divided into two exclusive clubs for men, in addition to the 2nd club which is shared by Czech Republic and Portugal. The women, by contrast, are distributed over 5 clubs, with just the bottom one being exclusive, consisting of Latvia and Lithuania. For both males and females, the three worst performing countries (Belarus, Russia and Ukraine) failed to converge into a club.

The pattern for males in the *oldest life expectancy category* is broadly similar to the previous category. Seventeen countries (Denmark fails to converge) fall into the first club, though are now joined by the Czech Republic, while two lower clubs form among the transition group: one with the Baltic and CEE countries (excluding Poland and Slovak Republic which don’t converge) and one comprising the three CIS countries. For women, once again there is greater diversity in life expectancy at 65. The OECD group forms 3 clubs, with Japan (expected life expectancy at 65 of 23.70 years) and France (22.72 years) breaking away from the core (second) group, with an average life expectancy of 21.28, while the OECD laggards of Netherlands, United Kingdom and United States are caught up by Poland, Estonia, Czech Republic and Hungary. Below that are two exclusive transition groups, defined again by their affiliation to the Baltic and CEE region on the one hand and the CIS region on the other. This female heterogeneity provides further evidence in support of the Vallin and Meslé (2004) hypothesis discussed previously.

### Results from the Recursive Convergence Analysis

To understand how and whether the transition countries themselves have become more heterogeneous over time we turn to the results of our recursive convergence analysis. The summary results are presented in “Appendix 2” (males) and “Appendix 3” (females), with the data in the tables listed according to life expectancy in 2010.^5^

#### Male Life Expectancy Patterns

The life expectancy at birth data shows clearly that, from the outset of transition in 1990, before the main market and political reform programmes had taken effect, the transition countries formed two distinctive clubs: the countries of CEE (except Hungary) that had not been Soviet republics and the countries of the FSU. By 2010, the initial two groups fragmented into 4 clubs with Estonia and Hungary entering periods of consistent divergence. The Czech Republic was a member of the western club; Poland, Slovak Republic and Bulgaria were in the top transition club; Lithuania and Latvia had permanently departed from the bottom group which comprised of Russia, Ukraine and Belarus—countries left in a long-term low equilibrium club.^6^

For life expectancy among males from age 15 to 65, there were also two clubs during the early 1990s, with the Czech Republic and Bulgaria diverging from the remaining transition countries. By 1995, three clubs had become established, with the Czech Republic joining the OECD-dominated club, the Slovak Republic and Poland joining Bulgaria in the top transition club, and the remaining countries left in the lowest club. As transition progressed, there is evidence that Hungary progressed towards the top transition club, while Estonia, Lithuania and Latvia moved ahead of Belarus, Ukraine and Russia, though the latter three countries—at the foot of the life expectancy league—did not converge.

For life expectancy at age 65, in 1990, there were already six countries (Poland, Estonia, Lithuania, Ukraine, Bulgaria and Belarus) within an OECD club, alongside Denmark, Great Britain and Ireland. While Lithuania remained in this club until 1999, the others had all fallen permanently behind by 1992, though Czech Republic progressed into the OECD group by 1996 and was in the leading club by 2010. The remaining transitional countries formed a single club until 2001, when the division into more and less successful clubs began, with the latter typically comprising of Russia, Belarus and Ukraine.

#### Female Life Expectancy patterns

It is noteworthy that for life expectancy at birth, until the mid-1990s, females formed one transition club and the divergence into multiple clubs only commenced from 1997. On the high equilibrium side, Czech Republic (a permanent member of the western group by 1998) Poland, Slovak Republic, Hungary and Lithuania (permanent members of the western group by 2001) split from the single transition group which existed through much of the early and mid-nineties. By 2010, the remaining countries were split into two lower groups, at the bottom yet again, were Ukraine and Russia, while above those poor performers, life expectancy in Belarus, Latvia and Bulgaria started to improve more rapidly, such that they formed their own club by 2008.

Life expectancy from age 15 to 65 exhibits some especially interesting patterns. Not only do Czech Republic and Slovak Republic join the OCED club but they were also above a western member (Denmark) in the rankings. Among the remaining transition countries, there was a lot of diversity and a degree of temporal swapping, but by the end of the period a familiar pattern had emerged, with Belarus, Russia and Ukraine, though not converging into a club, at the bottom of the rankings, Lithuania and Latvia in a club above those three, Estonia, Hungary and Bulgaria in the lowest OECD club, with Poland, Slovak Republic and Czech Republic appearing in progressively higher OECD dominated clubs.

For female life expectancy at age 65, there were initially two clubs with Poland, Lithuania and Belarus in the highest club. By the end of the period, the by now familiar pattern of divergence had become established with a lower group (Russia, Ukraine and Belarus), a middle group (Latvia, Lithuania, Bulgaria and Slovak Republic), and an upper group (Poland, Estonia, Czech Republic and Hungary).

#### Summary

The recursive analysis, using the initial conditions of 1959, demonstrates clearly that as the post-communist period has progressed, the group of transition countries themselves have become more heterogeneous over time. The analysis also demonstrates distinctive gender and age patterns and reinforces that none of the between or within region heterogeneity we see is due to mortality among the under 15s. For males, life expectancy among these countries started the transition period in two clubs: FSU and CEE; but by the end of the period, each of these had formed two smaller clubs: the worst performers were clearly Belarus, Russia and Ukraine, who were left behind by the Baltic States. However, in the top club, the Czech Republic had left the other CEE countries behind and had converged on western standards. For females, the picture is a little less clear. The transition countries were more homogenous during the 1990s, but clearly splinter in the 2000s with the CEE and part of the Baltic region diverging clearly from Russia, Ukraine, Belarus and perhaps also, separately, Latvia and Bulgaria.

## Conclusion

In this paper, we are the first to employ an empirical methodology developed by Phillips and Sul (2007, 2009) to identify patterns in life expectancy convergence, at different ages, between and within a set of OECD and transition countries during the past half century. In line with the literature, our descriptive analysis demonstrates that, over time, the variance in life expectancy by age and gender has increased. This is consistent with the idea that multiple life expectancy clubs (CIS, Baltic States and CEE) may have emerged, through a process of aggregate divergence, in the post-Communist era. At the same time, the region as a whole is shown to have been consistently diverging from the OECD region since the 1960s.

In applying the convergence club methodology, we examine these patterns from a more analytical perspective and make three core claims. First, we confirm, using a methodology which allows the data to select the groups, that the transition countries have not, in general, converged on western life expectancy achievements. Among our sample, the highest ranked clubs (with the persistent exception of the Czech Republic) tend to be almost entirely ‘western’, no matter the age or gender grouping. Accordingly, we observe that the clubs at the lower end of the life expectancy scale are dominated by the transition countries but, whereas the western countries tend to converge into 1, or at most 2, clubs; the transition countries diverge over this period into multiple clubs. This is consistent with the thesis that some countries in the transition region are stuck in low-level equilibria, while others are beginning to converge on the health dynamics of western advanced economies.

Second, we find strong gender and age group specifics. In terms of age, we find support for the Vallin and Meslé (2004) theory of divergence and convergence in advanced economies. That is, the inequalities in life expectancy that we observe within and between these countries are driven by the greater heterogeneity within older populations, which may reflect the challenges that advanced economies now face in dealing with older populations suffering from multiple morbidities. In terms of gender we find that female life expectancy clubs consistently outnumber their male counterparts, both for OECD and transition countries, and particularly for life expectancy at older ages. An interesting question for future research revolves around establishing the nature of this heterogeneity and the extent to which it reflects Vallin and Meslé type developments within the advanced OECD economies or whether it reflects some transition country-specific patterns.

Third, our recursive methodology allows us to understand the dynamics of club convergence within the transition region. In turn, this facilitates a more nuanced understanding of life expectancy groupings than methodologies based on a priori assumptions. For both males and females, there is divergence over time. For males, between 1990 and 2010, the number of clubs increases from two to four, comprising by 2010: the Czech Republic in an OECD group; Central European countries in a second group; the Baltic region, in a third group; and the CIS countries remaining laggards in the final group. While this is in line with the type of stylised assumption that informs a priori approaches, the female pattern is less so. For females, through the 1990s, there was broadly speaking one club, which fractured very rapidly after 1998, into multiple clubs—particularly at higher ages—and became increasingly interspersed with OECD countries. This is particularly interesting in the context of the work by Noymar and Van (2014), who find strong evidence that male and female life expectancy co-move in all countries. These differing patterns, in the evolution of male and female life expectancy, therefore call for further monitoring and investigation.

Finally, we reflect briefly on the limitations and strengths of the paper. First, our analysis is based on data for sub-samples of both the transition country region and the group of advanced economies. While this is not ideal, we argue that we do incorporate the major transition and OECD economies and the potential geopolitical groupings within them and that therefore our results are important. Second, unlike the methodology of Timonin et al. (2016), we are unable to decompose the country specific contributions to the club convergence that we observe. Related to this, we also do not weight the countries by their population size so that Latvia, for example, has equal weighting with Russia. However, we are more interested in identifying the country clubs, regardless of population weightings, in part at least so that we can compare our emergent results with the literature that does make country groupings based on a priori assumptions. Finally, we interpreted our finding that, according to the 15e0 classification, some of the least developed countries are in the same club as the most advanced countries as evidence of rapid success in lowering infant mortality in the poorer countries and the diminished scope for advanced countries to make further gains in this domain. However, at least part of the explanation may lie in the underreporting of early life mortality in parts of the Former Soviet Union during the 1960s and 1970s (Danilova et al. 2016)

Offsetting these limitations are two particular strengths of the paper: (1) we are the first to apply a methodology in this context which allows for life expectancy convergence patterns to be identified by the data and so the clubs which emerge are not defined by a set of prior beliefs but are a product of statistical testing. Second, by analysing life expectancy at four different ages, and using both the end of period convergence estimates and the recursive estimates, we can provide a much more nuanced analysis of the nature and dynamics of club convergence among these countries.

To conclude, understanding life expectancy patterns in the post-Communist region is a complex task. There is no one single correct way of either characterising or classifying the state of the art or the evolution of life expectancy patterns since 1990. Our convergence club approach, while not providing causal explanations, has teased out some of the subtleties that define this complex phenomenon and that are often overlooked by broad-brush empirical approaches. We have therefore added to the understanding of how and to what extent the transition countries are converging on (changing) OECD norms.

## Figures and Tables

**Table 3:**
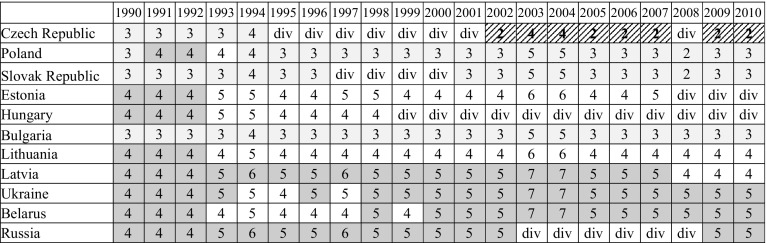
Recursive regression analysis for male life expectancy at birth

Dark grey cells indicate countries in the lowest transition club in that particular year; Light grey cells indicate transition countries in the highest life expectancy transition club; and the shaded cells, with bold typeface, indicate that the country belongs to a ‘western’ club

**Table 4:**
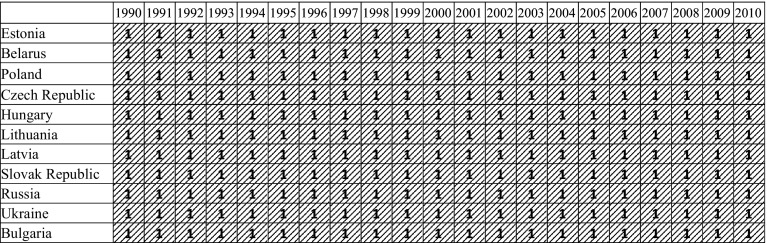
Recursive regression analysis for the average number of years lived by a male from age 0 to 15 (15e0)

Dark grey cells indicate countries in the lowest transition club in that particular year; Light grey cells indicate transition countries in the highest life expectancy transition club; and the shaded cells, with bold typeface, indicate that the country belongs to a ‘western’ club

**Table 5:**
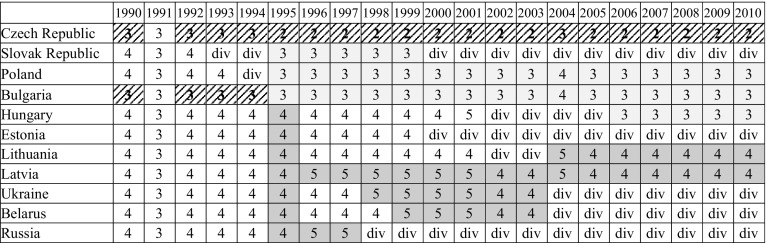
Recursive regression analysis for the average number of years lived by a male from age 15 to 65 (50e15)

Dark grey cells indicate countries in the lowest transition club in that particular year; Light grey cells indicate transition countries in the highest life expectancy transition club; and the shaded cells, with bold typeface, indicate that the country belongs to a ‘western’ club

**Table 6:**
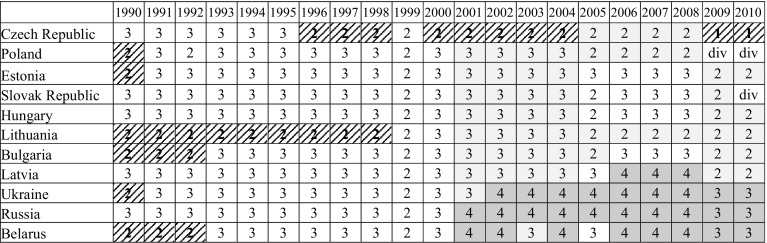
Recursive regression analysis for male life expectancy at age 65

Dark grey cells indicate countries in the lowest transition club in that particular year; Light grey cells indicate transition countries in the highest life expectancy transition club; and the shaded cells, with bold typeface, indicate that the country belongs to a ‘western’ club

**Table 7:**
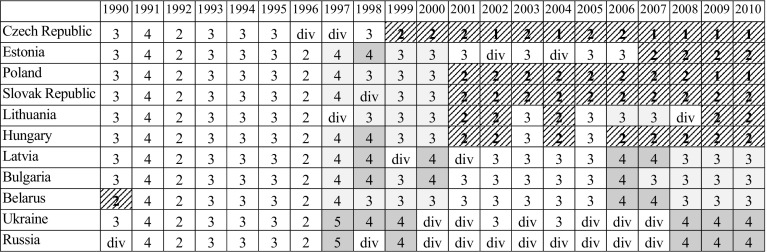
Recursive regression analysis for female life expectancy at birth

Dark grey cells indicate countries in the lowest transition club in that particular year; Light grey cells indicate transition countries in the highest life expectancy transition club; and the shaded cells, with bold typeface, indicate that the country belongs to a ‘western’ club

**Table 8:**
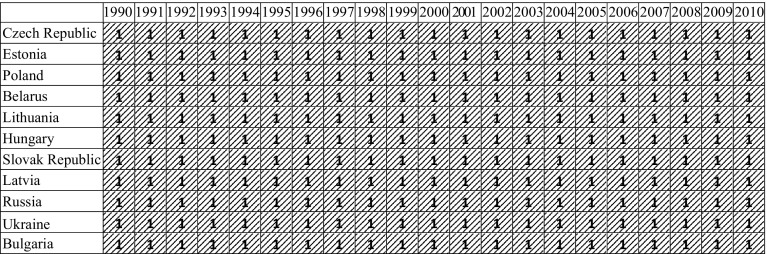
Recursive regression analysis for the average number of years lived by a female from age 0 to 15 (15e0)

Dark grey cells indicate countries in the lowest transition club in that particular year; Light grey cells indicate transition countries in the highest life expectancy transition club; and the shaded cells, with bold typeface, indicate that the country belongs to a ‘western’ club

**Table 9:**
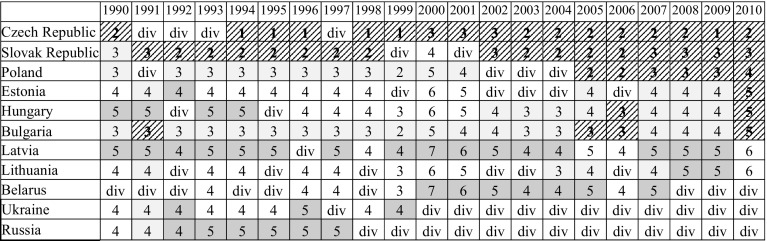
Recursive regression analysis for the average number of years lived by a female from age 15 to 65 (50e15)

Dark grey cells indicate countries in the lowest transition club in that particular year; Light grey cells indicate transition countries in the highest life expectancy transition club; and the shaded cells, with bold typeface, indicate that the country belongs to a ‘western’ club

**Table 10:**
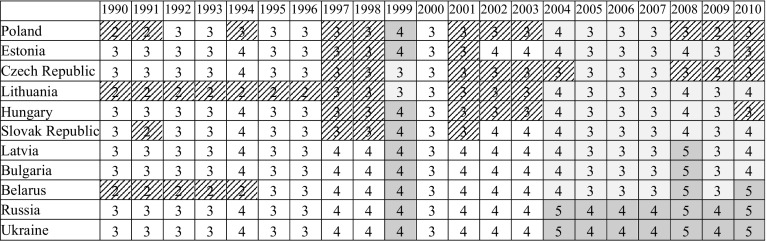
Recursive regression analysis for female life expectancy at age 65

Dark grey cells indicate countries in the lowest transition club in that particular year; Light grey cells indicate transition countries in the highest life expectancy transition club; and the shaded cells, with bold typeface, indicate that the country belongs to a ‘western’ club

## Appendix 1: The Log *t* Test Methodology

To formally identify the equilibrium ‘clubs’ in our data we employ a regression-based methodology (Phillips and Sul 2007) which examines whether the variance in the variable of interest across cross-sectional units decreases over time.

To capture the heterogeneous agent behaviour and model the heterogeneity in panels, data are usually decomposed according to a common factor structure and idiosyncratic effects. The simplest example is a single factor model:

1 $$X_{it} = \delta_{i} \mu_{t} + \varepsilon_{it} ,$$

where *X*_*it*_ is the observable variable of interest, *δ*_*it*_ represents unit specific factor loadings, *μ*_*t*_ the common factor and *ɛ*_*it*_the error term. By decomposing the variable of interest, *X*_*it*_, into a common factor *μ*_*t*_ and a time varying unit specific factor loadings *δ*_*it*_, Phillips and Sul (2007) offer the following representation of time varying factor model:

2 $$X_{it} = \delta_{it} \mu_{t}$$

where *δ*_*it*_ absorb*ɛ*_*it*_. If *μ*_*t*_ represents a common trend component in the panel of a country, *δ*_*it*_ represents the relative share in *μ*_*t*_ of country *i* at time *t*. Thus, *δ*_*it*_ is a measure of individual distance between the common trend component *μ*_*t*_ and *X*_*it*_. Phillips and Sul (2007) model the time-varying factor loadings *δ*_*it*_ in a semi-parametric manner, implying non-stationary transitional behaviour:

3 $$\delta_{it} = \delta_{i} + \sigma_{i} \xi_{it} L(t)^{ - 1} t^{ - \alpha } ,$$

in which, *δ*_*i*_ is fixed, *σ*_*i*_ is an idiosyncratic scale parameter, *ξ*_*it*_ is *i.i.d. (0, 1)* across *i* and weakly dependent over *t*, and *L(t)* is a slowly varying function (for example *L(t)* = *log t*), so that *L(t)* *→* *∞* as *t* *→* *∞,* and *α* denotes the speed of convergence. For all *α* ≥ 0 time-varying factor loadings *δ*_*it*_ converge to *δ*_*i*_. Convergence in the entire panel (the null hypothesis is $\delta_{it} \to \delta \;\;{\text{for}}\;{\text{some}}\;\delta \;{\text{as}}\;t\,\; \to \infty \;{\text{and }}\alpha \ge 0$) tests whether the distance between the common trend of *μ*_*t*_ and *X*_*it*_ for each country remains constant over time.

In order to model the transition coefficient *δ*_*it*_, a relative transition parameter *h*_*it*_ is constructed to measure *δ*_*it*_ in relation to the panel average at time *t* and to capture the relative departure of a country’s data series from the common growth path *μ*_*t*_:

4 $$h_{it} = \frac{{X_{it} }}{{\frac{1}{N}\sum\limits_{i = 1}^{N} {X_{it} } }} = \frac{{\delta_{it} }}{{\frac{1}{N}\sum\limits_{i = 1}^{N} {\delta_{it} } }}$$

If panel units converge $(\delta_{it} \to \delta {\text{ for all }}i{\text{ as }}t \to \infty \, )$ the relative transition parameters converge to 1 $(h_{it} \to 1{\text{ for all }}i{\text{ as }}t \to \infty \, )$ and in this case, the cross-sectional variance of *h*_*it*_, denoted by *H*_*t,*_ converges to zero:

5 $$H_{t} = \frac{1}{N}\sum\limits_{i - 1}^{N} {(h_{it} - 1)^{2} } \to 0 \, a{\text{s }}t \to \infty .$$

Phillips and Sul (2007) demonstrate that under convergence *H*_*t*_ has the limiting form

6 $$H_{t} \sim\frac{A}{{L(t)^{2} t^{2\alpha } }}{\text{ as }}t \to \infty {\text{ for some }}A > 0.$$

From here, it is possible to empirically evaluate the null hypothesis of convergence using a *log t* regression, implemented with the exclusion of *rT* observations which, from Monte Carlo simulations, Philips and Sul suggest is identified by the value of *r* = *0.3* for sample sizes below *T* = 50:

7 $$\log \left( {\frac{{H_{1} }}{{H_{t} }}} \right) - 2\log L(t) = a + b\log t + u ,$$

where *L(t)* = *log(t* + *1).* The fitted coefficient of log *t* is $\hat{b} = 2\hat{\alpha }$, where $\hat{\alpha }$ is the estimate of the speed of convergence. A one-sided t-tes is applied (Andrews (1991) and HAC (Heteroskedasticity and Autocorrelation Consistent) errors are used to test the inequality of the null hypothesis *α* ≥ 0. However, rejection of the null for the panel as a whole does not imply the absence of club convergence.

Phillips and Sul (2007) extend their own methodology and develop an algorithm for club convergence, which is based on a four-step procedure. This data-based identification of club members does not require an a priori specification of the number of clubs. First, cross-sectional units are sorted by the most recent time period observed in descending order. Second, subgroup *G*_*k*_ is formed using the first *k* units in the panel (k = 2,…, N) and the *log t* regression is run for each subgroup to calculate the convergence test statistic *t*_*k*_. The group with the highest and statistically significant (*t*_*k*_ > −*1.65*) *t*-statistic forms the core group. In the case that *t*_*k*_ > −*1.65* (5% significance) does not hold for the first two units in the sample, then the first unit is dropped and the procedure is repeated. If there are no such units for which *t*_*k*_ > −*1.65*, then there are no convergence clubs in the panel. Third, the *t*-statistic for the *log*-*t* regression is calculated by adding one unit at a time. A unit is a member of the club if this t-statistic exceeds some chosen critical value *c*. Phillips and Sul (2007) demonstrate that the recommended level for *c* is zero, because that reduces the risk of including a false member into a convergence club. If *t*_*k*_ > −*1.65*, this group forms a convergence club. Otherwise, the critical value *c* should be increased and the procedure repeated. If there are no units except the core group with *t*_*k*_ > −*1.65*, the convergence club consists only of the core group. Fourth, the remainder of the sample are then tested again to see if they form another club. If the rest of the sample diverges, convergence club methodology (steps 1–3) should be repeated for this sample.

Finally, Phillips and Sul (2007) suggest applying a smoothing technique to efficiently extract the long run component *X*_*it*_ in the presence of trend behaviour in life expectancy. In our empirical application we use the Hodrick–Prescott filter. The usual choices of the smoothing parameter (λ) for annual data are λ = 100 and λ = 400. Following Correia et al. (1992) we use λ = 400.^7^

In conclusion, we note that Phillips and Sul (2007, 2009) do not discuss the uniqueness of the convergence club solution. For example, it is possible to obtain a different solution by running a stepwise algorithm with sorting in the opposite order (from lower to upper life expectancies in our case). Instead, they emphasize the idea of the catching-up effect, when lagging countries catch up with more successful ones, which is realized through the logic of sorting from *t* the best state to the worst.

## Appendix 2: Recursive Convergence Analysis Results for Males (Only Transition Countries Presented)

See Tables 3, 4, 5 and 6.

## Appendix 3: Recursive Convergence Analysis Results for Females (Only Transition Countries Presented)

See Tables 7, 8, 9 and 10.

## Footnotes

1 The mortality belt comprises of Belarus, Estonia, Latvia, Lithuania, Moldova, Russian Federation and Ukraine.

2 Human Mortality Database, University of California, Berkeley and Max Planck Institute for Demographic Research (http://www.mortality.org/). Accessed on 25 June, 2017.

3 The calculation of 15e0 and 15e65 is respectively 15e0 = (T0 − T15)/l0) and 50e15 = (T15 − T65)/l15), where T(x) refers to the number of person-years remaining after exact age x (i.e. 0, 15, 65), and l(x) refers to the number of survivors at exact age x, assuming l(0) = 100,000.

4 Detailed category by category allocation procedures are available from the authors on request.

5 The full results, including for the OECD countries are available on request from the authors.

6 We don’t comment further on the results for the 15e0 age group because, as previously, for both men and women, all transition countries appeared in the western club throughout this period.
